# All Quiet on the TE Front? The Role of Chromatin in Transposable Element Silencing

**DOI:** 10.3390/cells11162501

**Published:** 2022-08-11

**Authors:** Luisa Di Stefano

**Affiliations:** Molecular, Cellular and Developmental Biology Department (MCD), Centre de Biologie Intégrative (CBI), University of Toulouse, CNRS, UPS, 31062 Toulouse, France; luisa.di-stefano@univ-tlse3.fr

**Keywords:** chromatin, transposable elements, transcriptional regulation

## Abstract

Transposable elements (TEs) are mobile genetic elements that constitute a sizeable portion of many eukaryotic genomes. Through their mobility, they represent a major source of genetic variation, and their activation can cause genetic instability and has been linked to aging, cancer and neurodegenerative diseases. Accordingly, tight regulation of TE transcription is necessary for normal development. Chromatin is at the heart of TE regulation; however, we still lack a comprehensive understanding of the precise role of chromatin marks in TE silencing and how chromatin marks are established and maintained at TE loci. In this review, I discuss evidence documenting the contribution of chromatin-associated proteins and histone marks in TE regulation across different species with an emphasis on Drosophila and mammalian systems.

## 1. Introduction

**Figure 1:**
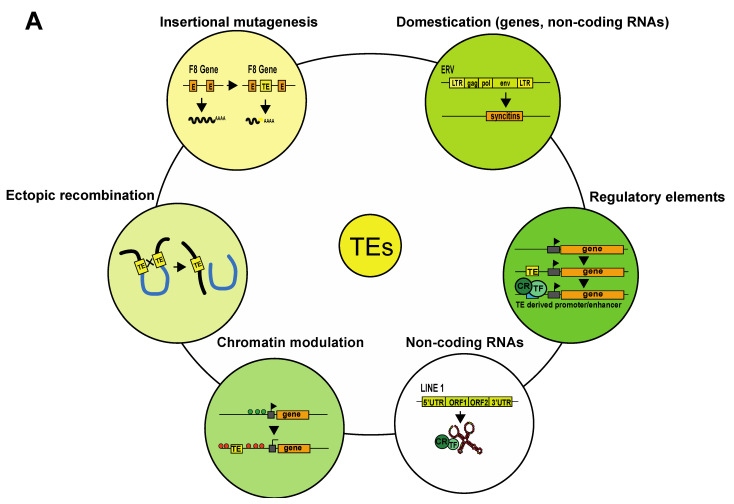

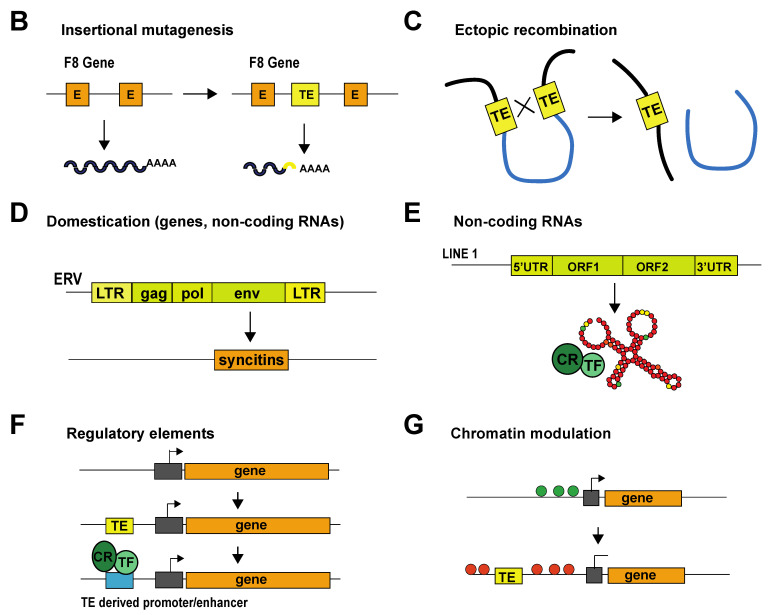
Impact of TEs on their host genome. (**A**) Examples of how TEs can impact genomes. (**B**) Schematic representation of a how insertion of a transposable element (TE) into the open reading frame of the coagulation factor VIII (*F8*) gene can induce insertional mutagenesis. This mutation was found in patients with hemophilia [1]. (**C**) Schematic representation of TE-induced ectopic recombination. (**D**) An example of TE domestication. Ancient env genes from ERVs have evolved into syncytin genes, which are involved in placenta formation [8]. Another example not represented here is that of *Rag1* and *Rag2*, which are involved in V(D)J somatic recombination in the immune system of vertebrates [18]. (**E**) An example of a TE transcript (LINE1) acting as an RNA scaffold for chromatin regulators and transcription factors. (**F**) TE sequences carry transcription factor binding sites, and their insertion can lead to novel gene-regulatory patterns in the host organism. (**G**) Example of how a TE can modulate chromatin by inducing the spread of heterochromatin. Abbreviations: E, exon; TE, transposable element; LTR, long terminal repeat; ORF, open reading frame; UTR, untranslated region; CR, chromatin regulator; TF, transcription factor.

Eukaryotic genomes are historical records of transposable element (TE) integration and mobilization events that occurred over millions of years. TEs and their remnants (degenerated TE sequences) represent a large fraction of eukaryotic genomes, constituting approximately half of the human genome. Transposable elements are mostly repetitive DNA sequences, and as their name indicates, they are capable of moving within the genome. Once considered “junk” DNA, it is now clear that TEs can both negatively and positively impact their host genomes (Figure 1A). TEs can threaten genomic stability through their ability to move around the genome; the insertion of a TE into a coding gene or a gene regulatory element impacts gene structure and expression and can lead to diseases such as cancer, hemophilia or neurodegenerative disorders (Figure 1B) [1,2]. TEs can also trigger chromosome deletions, duplications, inversions and translocations through ectopic recombination between TEs belonging to the same family [3] (Figure 1C). TE-driven genomic rearrangements have been responsible for major genomic expansions, and there is evidence that they have contributed to speciation [4,5,6]. Intact TEs can code for proteins that allow them to hop within the genome; however, most TE sequences degenerate over time and lose this ability [7]. Nevertheless, they can still play important roles in the host genome. Some have become host cell genes, a phenomenon known as TE domestication. This is the case of the *Syncytin* genes involved in placental development [8] (Figure 1A,D). In addition to producing coding transcripts, some TEs can be transcribed to produce non-coding RNAs. These non-coding RNAs can exert specific biological functions, as is the case of a transcript produced by the LINE-1 retrotransposon that works as an RNA scaffold during mouse early developmental stages [9] (Figure 1E). Additionally, a growing body of evidence shows that TE sequences have been co-opted to serve as regulatory elements to host genes. TEs carry cis-regulatory elements that, by duplication and insertion, can redistribute transcription factor binding sites and alter gene expression patterns. Epigenomic analyses indicate that a large fraction of mammalian regulatory binding sites (promoters/enhancers) have been provided by TE-derived sequences (Figure 1F) [10,11]. TE derived cis-regulatory elements can also influence chromatin architecture by serving as binding sites for CTCF (the CCCTC-binding factor), a sequence-specific DNA-binding protein that contributes to the establishment of chromatin loops [12,13]. Sometimes, TE presence itself can regulate host genome expression by modifying chromatin accessibility. For example, TEs can act as heterochromatin nucleation centers by inducing the spread of silencing marks from the TE to the adjacent cis-regulatory elements of host genes, thus inducing their repression (Figure 1G) [14]. However, TEs can also create de novo insulator regions, shielding a gene from heterochromatin expansion and allowing its expression [15,16,17]. 

In summary, TEs, through their capacity to impact gene expression patterns and induce genome instability, are an important source of genetic variation and a driving force of genomic evolution. Thus, understanding how TEs are regulated is a fundamental goal in biology. In this review, following a brief overview of TE classification, I focus on the current state of knowledge concerning the chromatin-based mechanisms of TE regulation with examples mainly taken from Drosophila, mice and humans. 

## 2. Classes of TEs

**Figure 2:**
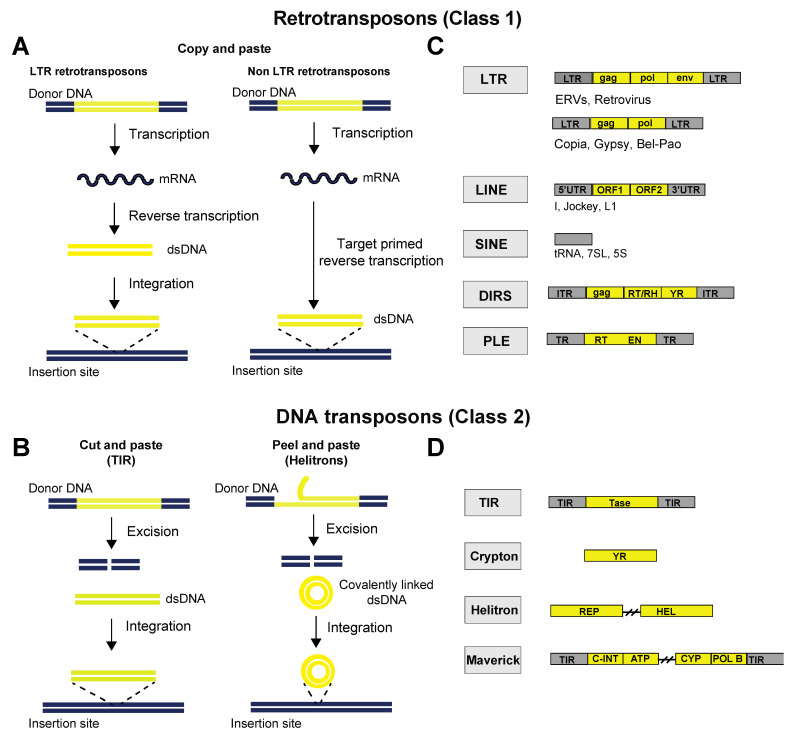
Schematic representation of the mobilization mechanisms of transposable elements. (**A**) Schematic representation of the “copy-and-paste” mobilization mechanism of retrotransposons. Retrotransposons replicate through an RNA intermediate and a reverse transcription step. LTR retrotransposons produce a double-stranded DNA (dsDNA) intermediate that integrates into a new locus, whereas non-LTR retrotransposons retrotranscribe directly at the target locus after cleaving genomic DNA, a process known as ‘target-primed reverse transcription’. (**B**) Schematic representation of the “cut-and-paste” and “peel-and-paste” mobilization mechanisms of DNA transposons. Both mobilization mechanisms require the excision of the transposon DNA from its original locus and its reintegration into another locus, but the “peel-and-paste” mechanism requires the formation of a circular double-stranded DNA (dsDNA) intermediate. The mechanism of replication of maverick and crypton elements has not been determined. (**C**,**D**) Classification of eukaryotic transposable elements (as proposed by Wicker et al. [21,22]). Genetic structures of representative transposable elements from each order. Yellow boxes represent open reading frames (ORFs), and grey boxes represent non-coding domains. Element lengths are not to scale. Abbreviations: LTR, long terminal repeat; ORF, open reading frame; UTR, untranslated region; ENV, envelope protein; GAG, capsid protein; RT, reverse transcriptase; RH, ribonuclease H domain; ITR, inverted terminal repeat; TR, terminal repeat; EN, endonuclease; YR, tyrosine recombinase; TIR, terminal inverted repeats; Tase, transposase; REP, replication initiator; Hel, helicase; C-INT, integrase; ATP, packaging ATPase; CYP, cysteine protease; POL B, DNA polymerase B.

Transposable elements are broadly classified on the basis of their mechanism of transposition as class I elements (retrotransposons) and class II elements (DNA transposons). Class I elements are transcribed into an RNA intermediate and use reverse transcriptase to form a new copy of their DNA, which is then inserted into the host genome (copy and paste) (Figure 2A). Class I elements are subdivided, on the basis of the presence or absence of long terminal repeats (LTRs), into LTR and non-LTR elements (Box 1). For LTR elements, the retrotranscription occurs in cytoplasmic virus-like particles, and the resulting dsDNA is then imported into the nucleus, where an integrase inserts it into the host genome. For non-LTR retrotransposons, retrotranscription occurs at the target locus of the host genome, a process known as ‘target-primed reverse transcription’ [19]. Class II elements encode a transposase enzyme that excises the parental sequence from a donor site and reintegrates it into another location in the genome (cut and paste) (Figure 2B). Therefore, in contrast to retrotransposons, they generally do not accumulate in copy number. However, they have adopted strategies to increase in copy number by transposing during host DNA synthesis from replicated to unreplicated sites or by taking advantage of the error-prone homologous recombination repair process [20]. More recently, rolling-circle elements (e.g., Helitrons) have been identified as a distinct group of abundant DNA transposons that do not replicate via the “cut-and-paste” mechanism but through a “peel-and-paste” mechanism. It has been hypothesized that the sense strand is “peeled” off, serving as a template to synthesize a second strand to form a circular double-stranded DNA (dsDNA) intermediate (Figure 2B) [20].

Independent from their mechanism of amplification, transcription is a crucial step in the replication of all groups of transposons. In the case of retrotransposons, RNA serves as a template for both the translation of TE proteins and for reverse transcription. For DNA transposons, transcription allows the expression of the transposase, which is essential for mobilization. These observations underline the need to fully understand the mechanisms underlying TE transcriptional regulation.

Box 1 **Classification of Transposable Elements.**

The classification of TEs is constantly being updated thanks to the development of novel tools that allow for a more refined TE classification and the discovery of new TE types. Traditionally, TEs have been classified into two classes on the basis of the DNA or RNA intermediate of their element: retrotransposons (class 1) and DNA transposons (class 2) (Figure 2A,B). Retrotransposons can be further classified into five orders based on their structural organization and mechanistic aspects of replication: long terminal repeats (LTRs), long interspersed nuclear elements (LINEs), short interspersed nuclear elements (SINEs), DIRS-like elements (DIRSs) and Penelope-like elements (PLEs) (Figure 2C). LTR elements are characterized by the presence of 5’ and 3’ non-coding long terminal repeat sequences that control the expression of retroviral genes. LINEs contain a 5’UTR and a polyA signal and encode all the proteins necessary for retrotransposition. SINEs are non-autonomous elements, the retrotransposition of which relies on functions coded by coexisting LINEs. DIRS-like elements have diverged from the other retrotransposons because they do not possess an integrase (INT) but rather use a tyrosine recombinase (YR) to integrate in the host genome. PLEs harbor an ORF coding for a protein that contains reverse transcriptase (RT) and endonuclease (EN) domains. PLEs are absent from mammalian genomes but can be found in some other eukaryotic genomes, including Drosophila, where they can cause hybrid dysgenesis syndrome, which is characterized by simultaneous mobilization of several unrelated TE families in the progeny of crosses involving different strains of the same species.

DNA transposons (class 2) are subdivided into the following orders: terminal inverted repeats (TIRs), Cryptons, Helitrons and Mavericks (Figure 2D). TIRs are characterized by the presence of terminal inverted repeats (TIRs) and encode a transposase that mediates excision and integration through binding to TIRs. Cryptons are simple transposons consisting in a single ORF coding for a tyrosine recombinase (YR). Helitrons code for a helicase. They replicate via the “peel-and-paste” mechanism by forming a circular double-stranded DNA (dsDNA) intermediate, earning the name of rolling-circle transposons. Mavericks are large DNA transposons encoding various proteins, including a DNA polymerase and an integrase.

## 3. TE Silencing

TE insertions and ectopic recombination between TEs can be harmful for the host genome. Additionally, independently of transposition, excessive TE transcription in the germline and in the soma can interfere with host cell function by activating the interferon response [23,24]. Therefore, eukaryotic genomes have developed a range of molecular mechanisms to silence TEs. Small RNAs are important tools to induce TE silencing in many organisms, including nematodes, plants, flies and yeasts. In eukaryotes, several classes of small RNAs associate with members of the Argonaute protein family to regulate gene and transposon expression both transcriptionally and post-transcriptionally. Whereas in flies and mammals, the importance of small-RNA-mediated TE silencing in somatic tissues is less clear, PIWI-interacting RNA (piRNAs), small noncoding RNA of 23−31 nucleotides (nt), play a pivotal role in TE silencing in the germline. Originally identified in Drosophila [25] but later found to be evolutionarily conserved, piRNAs originate from intergenic DNA elements known as piRNA clusters, which are rich in transposon sequences. piRNA precursors transcribed from these clusters are exported to the cytoplasm, where they undergo primary piRNA biogenesis [26,27,28,29,30,31,32,33,34], which consists of their endonucleolytic cleavage, followed by loading into Piwi protein. Three PIWI proteins have been described in Drosophila (Piwi, Aubergine (Aub) and Argonaute 3 (Ago3)) and mice (*Piwil1*, *Piwil2*, and *Piwil4*), whereas most primates have four PIWI genes (PIWIL1-4). The piRNAs loaded onto PIWI proteins undergo 3′ end processing and 2′-O-methylation at their 3′ termini [35,36,37]. In germline cells, in addition to primary piRNA biogenesis, a secondary piRNA biogenesis pathway known as the ‘Ping-Pong’ amplification loop amplifies the piRNA pool to achieve TE post-transcriptional silencing [38,39]

Mature piRNAs can suppress TE expression either post-transcriptionally by inducing the degradation of TE RNAs (post-transcriptional gene silencing (PTGS)) or transcriptionally (transcriptional gene silencing (TGS)) by driving PIWI to TE transcripts via RNA-RNA pairing and tethering chromatin modifiers that direct the deposition of repressive chromatin marks at TE loci. TE TGS has been found to operate in multiple organisms, including flies and mice [26,35,36,37,38,39,40,41]. 

Many excellent recent reviews describe how small RNAs can induce TE silencing [38,42,43,44]. In this review, after providing a brief overview of chromatin, I will focus on describing the proposed mechanisms by which chromatin could influence transcriptional TE silencing.

## 4. Chromatin 

Finely tuned transcription of transposable elements requires a dynamic regulation of chromatin structure, and the precise role of chromatin marks in this process is a subject of intense study. Chromatin is organized into structurally distinct domains called heterochromatin and euchromatin. Euchromatin is generally associated with active transcription and is characterized by an open, unfolded structure that ensures the accessibility of DNA to the transcriptional machinery, thereby favoring gene transcription. Heterochromatin is densely packed and mostly transcriptionally silenced. Heterochromatin can be further subdivided into constitutive heterochromatin, including gene-poor and repeat-rich regions proximal to telomeres and centromeres, and facultative heterochromatin, including regions interspersed within euchromatin that are silenced in a cell-type-specific manner. Heterochromatin plays critical roles in ensuring genome integrity by safeguarding mitotic fidelity, by preventing aberrant recombination between repetitive regions and by silencing the expression of transposons and satellite DNA [45]. 

Chromatin domains and subdomains of chromatin are distinguished by specific combinations of histone post-translational modifications (PTMs), DNA modifications and chromatin-bound factors (proteins and RNAs). At the molecular level, several mechanisms are in place to regulate chromatin, including addition and removal of DNA modifications or histone PTMs. Here, I will focus on those chromatin marks and factors that are currently associated with TE transcriptional regulation. 

### 4.1. Brief Overview of the Role of DNA Methylation in TE Silencing 

DNA methylation is a chemical modification that provides essential epigenetic information and has been implicated in TE silencing, genomic imprinting, X inactivation and regulation of gene expression [46]. Deregulation of DNA methylation results in embryonic abnormalities in mice and is a common feature of many cancer types [46]. 5-methylcytosine (5mC) is a widespread DNA modification present in many organisms, from bacteria to humans, often in the context of a CpG dinucleotide. However, some eukaryotes, including *Drosophila melanogaster*, *Caenorhabditis elegans* and *Saccharomyces cerevisiae*, either lack or have very low levels of 5mC [47], indicating that alternative mechanisms exist in these organisms to fulfill the role played by DNA methylation in vertebrates.

Cytosine methylation (5mC) occurs mostly in CpGs dinucleotides, which are abundant in repetitive sequences in intergenic regions, and in so-called CpG islands (CGIs), which are short stretches of CpGs nucleotides enriched in promoter regions. CGIs are generally refractory to DNA methylation, and, when present, methylation in CGIs strongly correlates with gene silencing [48]. Therefore, DNA methylation is broadly considered to be a repressive epigenetic mark. 

5mC is deposited by DNA methyltransferases (DNMTs), which, in eukaryotes, are classified as DNMT1 or DNMT3. DNMT1 preferentially methylates hemimethylated CpG dinucleotides following DNA replication, thus enabling maintenance of 5mC across cell division, whereas DNMT3-type enzymes catalyze de novo deposition of 5mC. 

Disruption of these enzymes has provided important information with respect to their role in TE silencing. In the plant *Arabidopsis thaliana*, in the fungus *Neurospora crassa* and in *Danio rerio* (zebrafish), loss of DNMT function results in accumulation of TE transcripts and increased transposition [49,50,51,52]. In *Dnmt1* knockout mice (KO), intracisternal a particle (IAP) retrotransposons, which are predominantly young and active endogenous retroviruses (ERVs), are highly derepressed [53]. Mutation of *Dmmt3C*, a methyltransferase specifically expressed in male fetal germ cells, results in activation of evolutionarily young families of retrotransposons [54]. Similarly, inactivation of a DNMT cofactor DNMT3L in mouse male germ cells results in reactivation of IAP and LINE1 retrotransposons and male sterility [55,56].

In mammalian somatic cells, DNA methylation of TEs is generally stably maintained. Nonetheless, retrotransposon activation has been observed in the brain and is correlated with reduced 5mC levels [57]. Furthermore, global DNA hypomethylation is a common feature of cancer cells and is associated with aging. Consistently, TEs were found to be reactivated in these contexts [58,59]. Whether TE activation associated with aging contributes to neurodegenerative disorders remains to be established. In cancer, TE expression and activation can have a double role. On one hand, TEs can induce new mutations by inserting themselves within oncogenes, tumor suppressor genes or their regulatory regions, thus altering their expression [55,60]. On the other hand, TE reactivation can elicit an immune response that leads to cell death and sensitizes tumor cells to immunotherapy [24,56]. Paradoxically, DNMT inhibitor (DNMTi) efficacy might be partly attributed to TE activation and consequent activation of the antiviral response, as DNMTi treatment has been shown to lead to an antiviral interferon response [23,61].

In addition to 5mC, other DNA modifications have been implicated in TE silencing, including N6-methyl adenosine (6mA) and N-4 methylcytosine, although their specific roles have not been fully established [62,63,64]. In this review, I will focus on the role of histone marks in TE silencing. To readers who would like to read more about the role of DNA methylation in TE silencing, here are some excellent and extensive reviews on the subject [46,65,66].

### 4.2. Histone Marks

Histones are subject to many kinds of post-translational modifications, the most extensively studied of which are acetylation, methylation, phosphorylation, ubiquitination and, more recently, SUMOylation. These chemical modifications confer specific properties to histones and contribute to either the opening or compaction of chromatin. Histone modifications are dynamically regulated by the activity of so-called “writer proteins”, which add histone marks, and “eraser proteins”, which remove the marks. Multiple writers and erasers with varying activities have been identified. Importantly, histone PTMs and DNA modifications serve as binding docks for “reader” domain-containing proteins that recognize these modifications. These reader proteins can carry additional domains capable of modifying chromatin, and/or they can reside in complexes with other proteins capable of adding or removing specific marks [67]. 

Histone lysine acetylation is associated with productive transcription. Adding an acetyl group to lysines adds a negative charge to the histone, thus reducing the interaction between histones and DNA. In addition, the acetylated lysines are recognized by different factors, including several bromo-domain-containing proteins that can actively remodel chromatin and regulate the recruitment of the transcriptional machinery [68]. Similarly, phosphorylation of serines and threonines opens chromatin by adding a negative charge to histones but also by evicting silencing complexes [69]. In contrast, histone methyl marks do not alter the charge of histones, and different methyl marks can be found either on actively transcribed (e.g., H3K4me3) or in silenced chromatin loci (e.g., H3K9me3). Spatial arrays of methylated histone lysines are thought to serve as a scaffold for the assembly of repressive and activating complexes. This is also the case for histone SUMOylation and ubiquitination, which were originally associated with silencing but that were later found to also act as signals to recruit activating complexes [70,71].

Other histone modifications have been reported, including crotonylation, butyrylation, propionylation, tyrosine hydroxylation, biotinylation, neddylation, O-GlcNAc, ADP ribosylation, N-formylation, proline isomerization and citrullination [72]. However, because their role in TE regulation is largely unknown, they will not be discussed in this review.

Crosstalk networks exist among histone marks and offer a means of leveraging desired, diversified outcomes. For example, the presence of one specific mark can favor or prevent the deposition of a second mark, and combinations of specific sets of marks (chromatin states) have been associated with specific functional outcomes [73]. The correlation between the presence of specific histone marks and the transcriptional status of a gene lead to the hypothesis that histone marks alone and in combination are the basis of a language or “code” that instructs changes in gene expression [74,75]. However, this language is quite complex, as various combinations of marks can result in similar outcomes, and each mark could be interpreted in different ways depending on the local chromatin environment, on the 3D folding of the genome and on the availability of effector proteins. This rich and complicated language generated by the diversity of histone PTMs confers the possibility of modulating and finetuning chromatin to achieve specific outcomes. The role played by histone PTMs in TE silencing is only starting to be dissected, and it will be discussed in the rest of this review.

### 4.3. Roles of Histone Marks

In addition to DNA methylation, histone modifications have also been shown to play a role in TE regulation. DNA methylation often overlaps with histone marks so that the two reinforce each other, ensuring stable TE repression [76,77]. However, certain classes of TEs seem to be relatively hypersensitive to the loss of either DNA or H3K9 methylation in specific developmental contexts. For example, in mouse embryonic stem cells, simultaneous knockdown of the three DNMTs only affects IAP expression, whereas individual depletion of the histone H3K9 methyltransferase SETDB1 (SET domain bifurcated 1) affects ERV expression [70,71]. Importantly, repressive histone marks might play an essential role in TE silencing when germ cells and embryos undergo phases of epigenetic reprogramming in which DNA is hypomethylated [78,79]. These early developmental phases coincide with a transient upregulation of TE transcription followed by a wave of repression. Whereas emerging evidence suggests that regulated TE expression is associated with normal embryogenesis, excessive and widespread TE deregulation can pose a risk with respect to the integrity of the genome [80,81]. Therefore, deposition of repressive histone marks could minimize the potentially negative consequences of TE derepression and activation in this particularly sensitive stage. Furthermore, histone methyl marks may play a prominent role in TE silencing in organisms where DNA methylation is low or absent, as is the case in Drosophila. 

### 4.4. Histone Methylation

**Figure 3:**
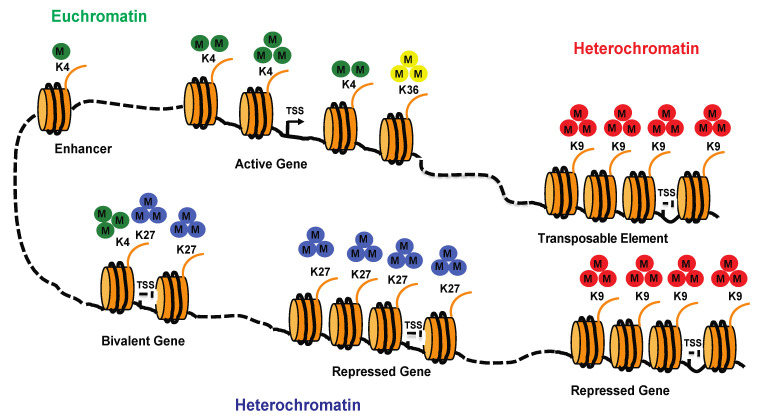
Modulation of chromatin organization and gene transcription by histone lysine methylation. A schematic representation of euchromatin and heterochromatin showing the main lysine methyl marks on histone H3 and their prevalent localization in the genome (euchromatin and heterochromatin). Abbreviations: K, lysine; TSS, transcriptional start site; M, methyl residue.

Histone methylation mainly occurs on lysine (K) and Arginine (R) residues. Histone methylation influences the transcription of genes by recruiting effector proteins, which in turn mediate the assembly of protein complexes that drive gene activation or silencing. Histone methylation is dynamic, and its tight regulation contributes to the coordinated expression of specific gene networks during normal development. The association of histone Arginine methylation with gene expression is poorly understood; in contrast, a strong correlation has been established between the state of methylation on specific histone lysines and gene transcription. Lysines can be mono-, di- or trimethylated, and depending on the degree of methylation, the residues affected and the chromatin context in which the methylation occurs, the presence of the methyl mark can have a positive or negative effect on transcription. H3K9 and H3K27 methylation is generally repressive; however, whereas H3K9me3 is abundant in pericentric heterochromatin and TEs, H3K27me3 is generally deposited in genes located in facultative heterochromatin and silenced in a cell-type-specific manner (Figure 3). In addition to H3K9me2/3 and H3K27me3, other histone marks are enriched in heterochromatin, including H4K20me3, H3K64me3 and H3K56me3 [45]. 

Euchromatin-enriched histone marks include acetylated lysines and methylated H3K4, H3K79 and H3K36. Genome-wide studies have shown that H3K4me3 is enriched at the transcriptional start site (TSS) of transcriptionally active genes with H3K4me2 and H3K4me1 just downstream, creating a gradient of H3K4 methylation [82] (Figure 3). H3K4me1 is also enriched in enhancers [83]. Methylated H3K79 and H3K36 are normally enriched in gene bodies [84]. Certain genes can harbor simultaneously “repressive” and “activating” marks in their regulatory regions (Figure 3). These so-called “bivalent domains” were discovered in lowly expressed developmental genes in embryonic stem cells and were found to be enriched in both H3K4me3 and H3K27me3 marks [85]. During cell differentiation, the loss of either the activating or the repressive mark primes the gene for either activation or repression, depending on the stimulus received [85]. This is a way to ensure that the appropriate genes are expressed in each tissue in a timely manner. 

### 4.5. H3K9 Methylation, a Defining Feature of TEs?

Histone 3 lysine 9 di and trimethylation (H3K9me2/3) is the hallmark of constitutive heterochromatin. H3K9 methylation is generally abundant in inactive genes and transposons. H3K9-decorated heterochromatin domains undergo profound rearrangement during development, and their dynamic regulation is essential for establishing and maintaining specific cell fates [86,87]. 

It has been proposed that H3K9me2/me3 induces silencing by serving as a binding site to recruit heterochromatin protein 1 (HP1) [88,89,90]. HP1 molecules multimerize and recruit additional chromatin factors to promote chromatin condensation and repression of transcription [91,92]. Nevertheless, some genes in heterochromatin can be actively transcribed despite the presence of H3K9 methylation in their gene body. It has been proposed that in these cases, H3K9 methylation marks act to suppress spurious transcription derived from TE promoters located in introns and flanking sequences [16]. In mammals and in plants, H3K9 methylation is often coupled with DNA methylation. DNA methyltransferases have been detected in shared complexes with H3K9 methyltransferases, and the two appear to mutually bolster their functions to ensure DNA inaccessibility [67]. 

In the fission yeast *S. pombe*, the establishment of H3K9 methylation requires the enzymatic activity of one histone methyltransferase (HMTase), the Clr4 protein [90]. However, in other organisms, including Drosophila and mammals, multiple H3K9 methyltransferases exist and share a highly conserved SET domain, which is responsible for their catalytic activity. In mammals, the known H3K9 methyltransferases are suppressor of variegation 3-9 enzymes (Suv39h1 and Suv39h2 in mammals, known as Su(var)3-9 in Drosophila), G9a, GLP (G9a-like protein), SetDB1 (SET domain bifurcated 1, known as Eggless in Drosophila) and SETDB2. Studies suggest that each enzyme is partly redundant with the other H3K9 HMTs, with a variable degree of redundancy depending on the family of TEs and the developmental timing [93]. However, some specificities exist, as Suv39h enzymes seem to preferentially catalyze H3K9 trimethylation in constitutive heterochromatin; G9a mostly mediates H3K9 dimethylation in euchromatin; and SetDB1, also known as Eggless in Drosophila; catalyzes histone 3 lysine 9 trimethylation in transposons. These conclusions are based on the fact that Suvar3–9 mutants in Drosophila, as well as double Suv39h1 and Suv39h2 loss in mammals, result in a drastic reduction in H3K9 me2/me3 levels but not H3K9me1 in pericentric heterochromatin [94,95] whereas KO of G9a in ES mouse cells results in a reduction in H3K9 methylation, mostly in euchromatin [96], and KO of SETDB1 in mouse ES cells and Drosophila ovarian somatic cells results in TE upregulation [71,97]. 

The activity of H3K9-HMT is counterbalanced by histone demethylases, which remove methyl marks from K9 residues. These so-called “erasers” include members of the Jumonji (JmjC)-domain-containing family, with JMJD2/KDM4 proteins acting on H3K9me2/me3 and JMJD1/KDM3 acting on H3K9me2/me1 [98,99,100]. Additionally, the lysine-specific demethylase 1 (LSD1/KDM1A) initially identified as an H3K4 demethylase, has been subsequently proposed to demethylate H3K9me2/me1 in certain contexts [101].

The existence of several enzymes targeting H3K9 complicates the analysis of the role of this modification in mammals, and only recently, a compound mutant for the known SET-domain H3K9 methyltrasferases (KMTs) (Suv39h1/Suv39h2, Eset1/Eset2 and G9a/Glp) was generated in mouse embryonic fibroblasts (MEFs) [93]. In these mutant cells, H3K9 methylation levels were undetectable, and heterochromatin organization was strongly affected. RNA-Seq experiments showed the derepression of multiple classes of retroelements. Interestingly, comparison of the compound mutants with mutants lacking the function of each set of paralogous H3K9 KMT suggests that distinct enzymes have both specific and overlapping functions, with, for example, the ERV1 family being more sensitive to Eset1 depletion and the ERVK family to G9 and Glp depletion [93]. 

In *C. elegans*, only two H3K9 methyltransferases are known (MET-2 and SET-25), and their compound mutation results in decreased H3K9 methylation, correlating with increased expression of a subset of transposons and satellite repeats. MET-2/SET-25 double mutants are viable, but they are sterile, and they are subject to increased genome instability [94,102]. Whereas MET-2 appears to be essential for the repression of satellite sequences, SET-25 represses a subset of DNA and RNA transposons [94].

Depleting histone methyltransferases to study transposon regulation, although useful, has limitations. Many of these KMTs have other substrates apart from histones, confounding the analysis of the biological contribution of the H3K9 methyl mark per se. Although mutating the H3K9 residue is difficult in metazoans due to the repetitive nature of their histone genes, generation of histone mutants is possible in some animal models and has provided insights into the role of H3K9 methylation in transcriptional silencing. For example, Drosophila H3K9R mutant flies display strongly reduced HP1 deposition in pericentric heterochromatin, as well as increased expression of transposable elements and piRNAs [95]. 

### 4.6. Role of the KRAB-Znf Family of Transcription Factors in the Recruitment of H3K9 Methyltransferases

Although H3K9-HMTs and HP1 proteins can bind DNA, it is believed that the specific recruitment of these enzymes at TE loci mostly relies on additional factors, such as small RNAs and transcription factors.

**Figure 4:**
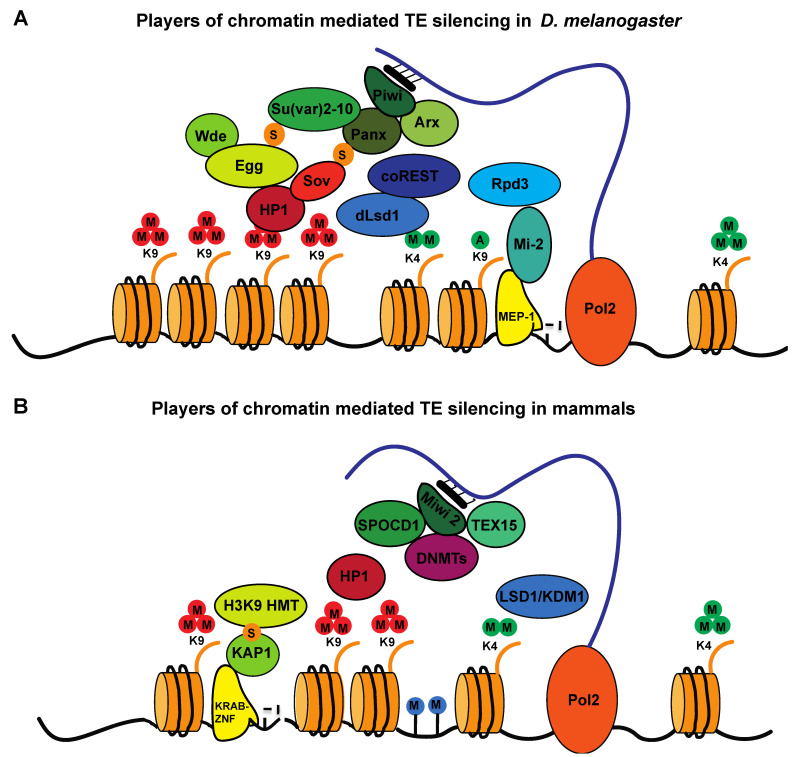
Models of chromatin-mediated transposon-silencing mechanisms. (**A**) Schematic representation of the factors implicated in TE silencing in Drosophila (**B**) and in mammals. Multiple mechanisms exist to repress TEs both in flies and in mice, including piRNA-directed silencing mediated by the Piwi/MIWI2-piRNAs complex, DNA methylation-dependent silencing mediated by DNMTs (in mammals), KDM1a-dependent histone demethylation and KRAB-ZNF-KAP1-mediated silencing. Whether these layers of control of TE silencing collaborate on the same TEs or act on different TEs or in different cell types/tissues remains to be fully elucidated.

Several members of the Krüppel-associated box zinc-finger protein (KRAB-Zfp) family of transcription factors have been shown to bind retrotransposon sequences in mice and humans [103,104,105,106]. It has been hypothesized that KRAB-Zfp proteins have evolved to recognize the different retrotransposons present in eukaryotic genomes [103]. Examples of KRAB-Zfps that bind retroelements are Zfp809 and Zfp708. These proteins, through their KRAB domain, are able to recruit the adaptor protein TRIM28/KAP1 (KRABS-associated protein 1), which, in turn, recruits SETDB1 [107,108,109,110,111]. In humans, SETDB1 recruitment by KAP1 is mediated by autoSUMOylation of its bromodomain [112]. SETDB1 dependent H3K9 methylation allows for the recruitment of HP1 and de novo DNA methylation [113] (Figure 4A). Another interaction partner of KAP1 is the human silencing hub (HUSH) complex (composed of TASOR, Mpp8 and periphilin 1), which recruits SETDB1 and an ATP-dependent chromatin remodeler, MORC2 [114,115], and was shown to silence evolutionarily young genes and retrotransposons [116]. A recent study suggests that the HUSH complex contributes to genome surveillance by silencing intronless invading DNA, including pseudogenes and retrogenes derived from recent transposition events [117].

### 4.7. piRNA-Dependent Recruitment of H3K9 Methyltransferases

Small-RNA-dependent recruitment of chromatin factors at TE loci constitutes an important mechanism of TE silencing in many organisms. It has been proposed that small RNAs might be necessary for the initial establishment of repressive H3K9 marks at TE loci both in germline and somatic cells [118,119]. In Drosophila, piRNA-loaded Piwi, in complex with Panoramix/Silencio (Panx) and Asterix (Arx), recognizes nascent TE transcripts via RNA–RNA pairing [35,97,120,121,122,123]. This piRNA-guided target recognition complex (Piwi/Panx/Arx) relies on the activity of the histone H3K9 methyltransferase Eggless (Egg) and its cofactor, Windei, on TE loci to promote TE silencing [97,120,124,125]. Additionally, it has been shown that SUMOylation by the SUMO E3 ligase Su(var)2-10 is also important for TE silencing, and it has been proposed that Su(var)2-10 recruits the histone H3K9 methyltransferase Eggless (Egg) and its cofactor, Windei, to TE loci, thus promoting TE silencing (Figure 4A) [16]. Recent data also show that Panx is SUMOylated, although in a Su(var)2-10-independent manner, and that Panx SUMOylation, which is Piwi-dependent, is required for its interaction with the zinc finger protein small ovary, a factor implicated in heterochromatin formation [126]. However, specific depletion of Piwi in the nucleus results in a reduction in H3K9 methylation that is limited to a subset of TE families, indicating that Piwi-independent mechanisms exist [127]. Interestingly, Piwi depletion also results in an increase in the activating H3K4me2 mark at some TE loci [127], suggesting that Piwi represses a subset of TEs through association with protein complexes responsible for maintaining low levels of H3K4me2. This possibility is supported by an involvement of the histone demethylase dLsd1 in TE silencing. This demethylase acts on H3K4me2 residues, and depletion of dLsd1 activity has recently been shown to provoke an increase in the transcription of many TE families in the ovaries, both in the germline and in somatic cells [128,129]. Additionally, repression of a reporter construct by artificial tethering of Panoramix is impaired by knockdown of dLsd1 and its cofactor, coREST [120]. Furthermore, the histone deacetylase Rpd3, a known partner of coREST, has been implicated in TE silencing, together with the chromatin remodeler mi-2 and the zinc-finger transcription factor MEP-1 [130]. All these factors have been shown to physically interact with components of the piRNA-guided target recognition complex in Drosophila [128,130]. Therefore, a model to achieve silencing of at least some TE loci could rely on the sequential activity of multiple chromatin factors, whereby dLsd1 would demethylate H3K4me2, which could in turn allow for H3K9 deacetylation by Rpd3 and subsequent H3K9 methylation by Egg. Such a scenario would limit the level of activating marks and increase the levels of repressive marks, potentially promoting TE silencing. It will be interesting to determine the hierarchy of events that leads to silencing and to determine whether all these factors are recruited by the piRNA-guided target recognition complex. Additionally, multiple recent studies implicate Nfx2, a paralog of the nuclear export factor Nfx1, in Panoramix-mediated, piRNA-guided TE silencing. Nfx2-containing complexes were named either SFiNX (silencing factor interacting nuclear export variant), PICTS (Panx-induced cotranscriptional silencing), PPNP (Piwi–Panx–Nxf2– P15) or Pandas (Panx–Nxf2 dependent TAP silencing) by the four groups that reported them [131,132,133,134]. More recently, the dynein light-chain LC8/Cut-up was shown to interact with Panx and to drive the dimerization of the PICTS complex. The authors also showed that Cut-up is essential for Panx-dependent TE silencing [135]. Further investigations will be required to determine the precise roles of these factors in TE silencing and heterochromatin formation. 

In mouse germline cells, piRNA-loaded MIWI2, in addition to allowing the deposition of repressive chromatin marks, also allows de novo DNA methylation [26]. It has been proposed that MIWI2 recruits DNMTs at TE loci (Figure 4B), but the mechanisms remain to be elucidated. Two recent papers shed some light on the mechanisms by showing that in the mouse male germline, two MIWI2-associated factors, SPOCD1 and TEX15, are required for de novo DNA methylation of a subset of TEs [136,137].

In summary, a feature of TE silencing pathways in multiple species is that heterochromatin formation is initiated by recognition of nascent transcripts by a PIWI family protein bound to a small RNA. Thus, nascent TE transcripts contribute to TE silencing. Interestingly, although both TE sequences and piRNA clusters are marked by H3K9 methylation, TEs are silenced, whereas piRNA clusters produce piRNA precursors. In Drosophila, the different outcomes (silencing vs. transcription) could be due to the fact that H3K9 methyl marks are read by HP1 at TEs and by Rhino, an HP1 paralog, at a subset of piRNA clusters [138,139,140]. Another layer of control could be at the level of nuclear export, because whereas piRNA precursor are normally exported in the cytoplasm [141,142], a study showed that TE transcripts export is inhibited by Nxf2 [131]. In any case, it remains to be determined to what extent H3K9 methylation is a cause versus a consequence of TE silencing. Studies have shown that the presence of H3K9 methylation does not always preclude transcription, especially when located outside of the TSS, and some heterochromatic genes require H3K9 methylation for their proper expression [35,143]. Moreover, genome-wide studies have revealed that TEs are marked by a complex pattern of chromatin modifications, including H4K20, H3K27 and H4R3 methylation; histone biotinylation and sumoylation; and the deposition of H3.3 variants [144]. 

### 4.8. H3K27 Methylation, an Ancestral form of TE Silencing?

H3K27 methylation is deposited by the catalytic subunits of the polycomb repressive complex 2 (PRC2) (EZH1 and EZH2 in humans), which methylate K27 [145] through their SET domains. The PRC2 complex is crucial for maintenance of stable differentiation [146], and mutations of PRC2 components have been identified in a variety of human cancers [147]. Whereas H3K27 methyl marks are associated with silent protein-coding genes and are a key component of facultative heterochromatin, they are not abundant at TE loci. However, PRC2 and H3K27methyl marks can be relocalized to repeat regions when DNA methylation and/or H3K9 methylation are perturbed either through mutation of writers or of readers of these marks or during early mammalian developmental, when DNA hypomethylation occurs naturally [78,148]. Therefore, H3K27me3 could be used as a backup mark for TE silencing in situations where H3K9 methylation and/or DNA methylation are impaired [149]. Interestingly, TEs of some ancestral eukaryotes, including ciliates and bryophytes, are marked by the presence of H3K27 methylation domains [149]. Similarly, in *Paramecium tetraurelia*, H3K27 and H3K9 methyl marks coexist in multiple TE families, and the ortholog of Ezh2, Ezl1, is implicated in their silencing [150]. Ezl1 interaction with components of small RNA/RNAi machinery appears to be responsible for its targeting of TEs [151]. It has been hypothesized that H3K27 methylation is an ancestral mechanism of TE silencing that has been largely replaced by a more stable silencing by H3K9 methylation and DNA methylation at the onset of multicellularization when the disadvantages of TE activation outweigh their possible advantages, especially in the germline. H3K27 methylation could still play a role in some TEs in mammals, as Leeb and colleagues showed that IAP and MLV elements are derepressed in double-knockout mouse ES cells for a component of the PRC1 and for a component of the PRC2 complex [152].

### 4.9. Role of Histone 4 in TE Silencing

Trimethylated histone H4 lysine 20 (H4K20me3) is enriched in heterochromatin, and H4K20me3 peaks are associated with LINEs, ERVs, satellite DNA and low-complexity repeats in human sperm and somatic (K562) cells [153]. In mice, H4K20me3 is catalyzed by the activity of SUV420H1 and SUV420H2 enzymes, and knockout of SUV420H2 in mouse ES cells results in derepression of repetitive DNA elements [154]. H4K20me3 is often found to co-occur with H3K9 methylation and DNA methylation in repetitive elements [144,155,156]. It has been proposed that H3K9 methylation acts upstream of H4K20 methylation, as depletion of K9 histone methyltransferases prevents the trimethylation of H4K20 by SUV420H1 and SUV420H2 in repetitive regions [71,157,158]. 

However, at specific loci, H4K20me3 can occur independently of H3K9me3, as is the case for the Charlie DNA transposon family in mouse ES cells, IAP retrotransposons in quiescent cells and in young DNA transposon subfamilies in early developmental stages of Xenopus tropicalis [159]. An independent study showed that the DNA methylase DNMT1 directly recognizes the H4K20me3 mark via its first bromo-adjacent homology domain (DNMT1_BAH1_), and this association potentiates the enzymatic activation of DNMT1 and could stabilize the repression of the LINE1 element [156].

H4K20 methylation is not the only H4 modification implicated in TE silencing. ChIP-Seq analysis in murine embryonic stem cells (ESCs) revealed an enrichment in dimethylation of arginine 3 on histone 4 (H4R3me2), a mark associated with transcriptional repression in LINEs, SINEs and LTR transposons [144,160]. Consistently, conditional loss of the protein arginine methyltransferase 5 (PRMT5), which is responsible for H4R3 methylation, in primordial germ cells (PGCs) causes upregulation of LINE1 and IAP transposons. It is important to highlight that PRMT5 depletion in PGCs also results in sterility and activation of a DNA damage response [161]. The authors proposed that PRMT5 is involved in guaranteeing transposon silencing and maintenance of genome integrity at times when the DNA is hypomethylated [161].

### 4.10. Sumoylation

Small ubiquitin-like modifier (SUMO) is a highly conserved, ubiquitin-like small protein. SUMO covalent attachment to target proteins modulates their function. SUMO is added to its targets through a conjugation cascade implemented by the activities of E1 (activating), E2 (conjugating) and E3 (ligase) enzymes [162]. This post-translational modification (PTM) can be reversed by SUMO-specific proteases [163].

SUMOylation has been shown to play a role in the formation of heterochromatin from yeast to mammals [112,164,165,166,167,168,169], and a genome-wide screen in Drosophila identified components of the SUMO pathway as factors required for TE repression [122]. In addition, it has been shown that auto SUMOylation of KAP1 in humans and Su(var)2–0 in Drosophila is required for SetDB1 recruitment in TEs. Additional SUMOylated proteins beyond KAP1 and Su(var)2–10 might also be required for SetDB1 recruitment and/or for TE repression. Accordingly, the “SUMO spray” hypothesis posits that SUMOylation of multiple proteins containing the rather common SUMOylation consensus sequence (including histones) could collectively contribute to the recruitment and maintenance of repressive effector complexes in TEs [16,170]. This hypothesis is in line with recent findings demonstrating that chromatin bound Panx is SUMOylated in a Piwi-dependent manner and that Panx SUMOylation is required for its interaction with the corepressor factor small ovary (Sov) [126].

### 4.11. “Active” Histone Marks at TE Loci

TEs are not universally repressed, as shown by an increasing number of studies revealing that TEs can be activated in stage-specific patterns [81,171]. For example, in the earliest stages of mouse development (from zygote to blastocyst stage), many retrotransposons are actively transcribed and contribute to embryonic development [9,172]. In the human and Drosophila brain, some retrotransposons are not only expressed, but they can also actively transpose, and it has been proposed that they contribute to the diversification of neuronal cell populations [173,174]. Additionally, many TEs have been shown to be reactivated under pathological conditions, including cancer [175,176,177]. However, the mechanisms underlying their dynamic regulation remain largely unknown.

Analysis of ChIP-Seq data from mouse ESCs shows that whereas TEs harbor repressive marks, they can also be labeled by a wide array of active marks, including histone acetylation, as well as H3K4me1 and me3 [144]. Quantification of the levels of the various chromatin marks at TEs in different human tissues and developmental stages shows that although a median of only 8% of TEs are in an active state in each epigenome, on average, 49% of TEs can be in an active state in at least one genome. Certain classes of TEs (notably, SINEs), certain tissues (brain and blood) and cancer cell lines are enriched relative to other classes/tissues for active marks [178]. Interestingly, ChIP-Seq data in ESCs show that repressive and active marks coexist for some TEs [144]. Although this coexistence could be an artifact reflecting the difficulty of mapping reads to a specific TE within a repetitive family, analyses of unique regions close to TEs obtained by qPCR amplification indicate that H3K9me3 and H3K27ac do coexist at some of the loci tested. Re-ChIP would be needed to verify whether the coexistence of these marks is due to a pooling effect, as the ChIP-Seq data were obtained from pooled cells. If confirmed, the coexistence of repressive and activating marks, the so-called bivalent domains, in some TEs in ESCs cells might mean that these TEs are kept in a poised state to be either quickly activated or repressed depending on the context [144]. Intriguingly, ChIP analysis of H3K9me3 and H3K4me3 in early mouse embryogenesis shows that these two marks are enriched in IAPs—LINE-1 and SINE B2—in the two-cell stage, whereas by the eight-cell stage, H3K4me3 levels have decreased and H3K9me3 levels remain unchanged [179]. Given that these TEs are highly expressed in the two-cell stage and their expression declines in the eight-cell stage, the authors suggest that their silencing in this developmental stage is a consequence of the loss of the activating marks rather than to the acquisition of H3K9 methyl marks [81]. These data thus suggest that the expression of some repetitive elements may be regulated by methylation and demethylation of H3K4. 

### 4.12. H3K4 Methylation 

H3K4 methylation is a mark that strongly correlates with active transcription. In budding yeast, a single SET1 methyltransferase is charged with all H3K4 methylation. In contrast, Drosophila possesses three H3K4 HMTs (dSET1, TRX and TRR), whereas further expansion in vertebrates has resulted in six H3K4 HMTs (SETD1A, SETD1B, MLL1, MLL2, MLL3 and MLL4). These enzymes play important and non-redundant roles during development, and many of them have been implicated in human diseases [69,180]. The enzymes responsible for removing the methyl marks from H3K4 are the histone demethylase KDM5 and LSD1, which remove H3K4me3/me2 marks and H3K4me2/me1 marks, respectively. Studies in a variety organisms suggest an important role of LSD1 in TE silencing. Transcriptomics analysis performed in murine ES cells and in Drosophila ovaries showed that KDM1A/dLsd1 depletion leads to increased expression of transposable elements [128,129,181]. Upregulation of transposable elements is associated with an increase in H3K4 methylation at target TEs [128,181], suggesting that the catalytic activity of KDM1A might be required for TE silencing. Furthermore, KDM1A/LSD1 was shown to physically interact with two important players of TE silencing, KAP1 in mice [181] and Piwi in Drosophila, although it remains to be established whether the interaction between Piwi and dLsd1 is direct [128]. Another study in mice showed that KDM1A null oocytes give rise to zygotes that are arrested by the two-cell stage and that this arrest is accompanied by perturbation in the expression of retrotransposons [182]. Importantly, in human cells, KDM1A inhibition causes TE reactivation, which in turn triggers an immune response that renders cancer cells more susceptible to immunotherapy [24]. Together, these data raise the possibility that active demethylation of H3K4 marks by LSD1 is required for silencing of transposable elements. 

Conversely, depletion of the H3K4 methyltransferase MLL2 in mouse ES cells results in a decrease in H3K4me3 in a subset of young L1 subfamilies. This catalytic activity seems to be required for expression of these young L1s, as overexpression of enzymatic dead MLL2 results in downregulation of their expression [183].

### 4.13. H3K36 Methylation

H3K36me3 is enriched in gene bodies, where it is thought to prevent cryptic transcription, whereas H3K36me2 is mainly found at TSS and intergenic regions and is regarded as an activating mark [69]. However, one recent study in Drosophila showed that large H3K36me2 domains are present in pericentromeric regions enriched for TE sequences and that H3.3K27M and H3.3K36M mutations cause a redistribution of H3K36 methylation marks away from transposon-rich regions, as well as deregulation of TEs [184]. This work raises the intriguing possibility that H3K36me2 could act as a repressive mark at TE loci, either directly or indirectly, through crosstalk with the heterochromatin machinery.

### 4.14. H3.3

H3.3 is a variant of the canonical H3.1 and H3.2 histone and differs from them only by four or five amino acids. In contrast to H3.1 and H3.2, which can only be incorporated into chromatin in the S phase, H3.3 can be deposited throughout the cell cycle [185]. H3.3 has been generally associated with gene activation. However, in mouse embryonic stem cells, H3.3 localization is not limited to euchromatic genes but intersects with H3K9me3-marked ERVs. Importantly, in H3.3 KO ES cells, the levels of H3K9me3 are reduced in TEs normally harboring H3.3 and correlate with derepression of their adjacent genes and with activation of IAP [186]. The authors propose that H3.3 deposition at TE loci precedes H3K9 methylation and contributes to the silencing of a subset of TEs [186].

### 4.15. Histone Acetylation

Histone acetylation is a hallmark of transcription, as it is widely correlated with actively expressed genes; however, genome studies show that some transposable elements can also carry histone acetyl marks [144,178]. For example, ChIP-Seq analysis of H3K9ac distribution in *A. thaliana* revealed that although the majority of the signal was located in actively expressed genes, approximately 300 TEs were marked by H3K9 acetylation [187]. In Arabidopsis, this mark is removed by HDA6, a histone deacetylase that has been implicated in TE silencing [188,189]. Similarly, the Drosophila histone deacetylase Rpd3 has also been shown to contribute to TE silencing [130]. Interestingly, treatment with the HDAC inhibitor valproic acid (VPA) increases the chromatin accessibility of SINE elements in the adult visual cortex of mice [190]. Although the data are still sparse, it is plausible that histone acetylation would mark active transposons in specific developmental times, such as in preimplantation embryos when some TEs are actively transcribed. Furthermore, the implication of HDACs and other “erasers” in TE silencing indicates that, at least in some cases, TE silencing needs to be actively maintained through the continuous removal of active marks.

### 4.16. The Emerging Role of Nuclear Architecture

Chromosome conformational capture and microscopy-based techniques have revealed that active and inactive chromatin domains tend to separate in space. Heterochromatin regions tend to cluster, forming tridimensional structures defined as “B” compartments, whereas active regions segregate in space into “A” compartments [191,192]. Within these compartments, chromatin folds into smaller domains that preferentially interact with themselves, commonly defined as topologically associating domains (TADs) [191,192].

The three-dimensional (3D) structure of the genome can influence gene expression, but its specific role in TE regulation has received little attention to date.

High-throughput chromosome conformation capture (Hi-C) in Drosophila ovarian cells depleted of Piwi shows that whereas long-range contacts are minimally affected by Piwi KD, short-range intra-TAD interactions in a subset of piRNA-targeted TEs are strongly decreased [193]. Similar results were obtained upon knockdown of Piwi cofactor Nxf-2 [193]. Using a tethering system to monitor Piwi–piRNA-mediated reporter silencing in ovarian cells, the authors showed that the increase in H3K9me3 and H1 mark is preceded by a decrease in H3K27 acetylation and H3K4me3 levels. Similarly, they observed that changes in nuclear localization precedes changes in chromatin conformation [193]. Therefore, they propose that Piwi–piRNA-mediated TE silencing occurs in a stepwise manner, whereby removal of active histone marks and relocalization within the nucleus is followed by an increase in repressive histone marks and chromatin conformation changes, thus proposing that Piwi triggers spatial regulation of TE loci [193].

Additionally, TEs contain functional regulatory sequences that could impact chromatin folding. These include binding sites for CTCF, which has a known role in chromatin loop and domain boundary formation. A recent paper provides evidence that in humans and mice, CTCF sites derived from TEs contribute to loop formation and that deleting two of these TEs in human cell lines eliminates these loops [194]. Based on an example in which TE deletion results in a loop shift to an alternative, ancient TE-derived CTCF site nearby, the authors argue that TE transposition could provide redundant CTCF motifs to assure the stability and robustness of 3D folding [194]. Confirming this intriguing possibility will likely require directed mutation of TE-derived CTCF sites.

### 4.17. Interplay between m^6^A RNA and Chromatin at TE loci

Similarly to DNA and protein, RNA can be modified by distinct types of modifications. One such modification, N^6^ methyladenosine (m^6^A), was recently linked to TE regulation. m^6^A is a modified base that is present in many coding and non-coding RNAs, predominantly at stop codons and 3′UTRs [195,196,197]. The presence of the m^6^A mark has been shown to influence gene expression and to play a role in early development and in cancer [198]. At the molecular level, this modification is co-transcriptionally deposited by methyltransferase-like 3 (MTTL3) in complex with its cofactor, methyltransferase-like 14 (METTL14), or by methyltransferase-like 16 (MTTL16) [199,200,201]. More recently, another enzyme, methyltransferase ZCCHC4, was shown to be able to deposit this mark [202]. Conversely, m^6^A can be erased by the m^6^A demethylase fat mass and obesity-associated protein (FCO) [203,204] or AlkB homolog 5 (ALKBH5) [205]. Additionally, many proteins are able to read this modification, including the YT521-B homology (YTH) family proteins and insulin-like growth factor-2 mRNA-binding protein (IGF2BP) family proteins [206].

Genome-wide analysis of METTL3 localization in mouse embryonic stem cells showed that METTL3 primarily localizes in heterochromatin and that it is enriched in IAP retroelements [207]. The authors then generated *Mettl3* KO cells and observed that these cells featured significant decreases in H3K9me3 and H4K20me3 on IAP elements, which correlated with increased transcript levels. This increase was not due to altered RNA stability but, rather, to chromatin changes [207]. Consistently, Mettl3 interacts with SetDB1 and TRIM28. An independent study by Chelmicki et al. also reported significant upregulation of IAP transcripts upon acute METTL3 and METTL14 degradation; however, the authors did not observe changes in chromatin marks at IAP loci in the short term [208]. Both studies revealed that IAP transcripts carry m^6^A marks recognized by Ythd-domain-containing proteins, and Chelmicki et al. showed that m^6^A marks decrease IAP transcript stability [208]. Another study showed that Ythdc1 KO in mESC cells also results in TE upregulation [209]. Through a series of genome-wide approaches (RIP-Seq, ChIP-Seq and ChiRP-Seq), Liu and colleagues found Ythdc1 to be enriched in retrotransposons, such as IAP and LINE1, with concurrent enrichment of H3K9me3, m6A marks and SETDB1 at these loci [209]. These epigenomic analyses and other biochemical evidence suggest that Ythdc1 mediates the establishment of H3K9me3 marks at TEs through its binding to m^6^A-modified LINE1 transcripts. [209,210]. In addition, Liu et al. and Chen et al. independently showed that Ythdc1 loss affects the transcription of genes implicated in the two-cell embryo (2C) program [209,210] through m^6^A-modified, LINE1-dependent silencing of the Dux locus, a transcription factor-coding gene, which is essential for two-cell fate and which was previously shown to be regulated by LINE1 RNA [9]. 

Although some discrepancies between the studies need to be resolved, globally available evidence indicates that m6A-modified transcripts derived from TEs can influence TE expression and heterochromatin deposition both in cis and in trans contexts. These recent findings pave the way to a more detailed and mechanistic study of the role of “epitranscripts” in the control of gene and TE expression.

## 5. Summary, Significance and Future Directions

In summary, it is clear that chromatin plays a significant role in the regulation of TEs. Nevertheless, many aspects of this regulation remain to be identified. One important point to address is to what extent histone marks play a causal role in TE silencing/activation. For example, although several studies in multiple organisms show that H3K9 methylation is a hallmark of TEs, some studies show that H3K9 methylation is not sufficient to ensure silencing of TEs and may not always be required. The precise cascade of events that leads to TE silencing in different contexts and the mechanisms responsible for the maintenance of this silencing and for their transgenerational inheritance remain elusive. Another important mechanistic question concerns the dynamics of TE regulation during development and in adult cells: which classes of TEs are expressed and in which developmental/pathological contexts? What is their biological role? How exactly are they dynamically regulated, and how is TE silencing maintained? Because nascent TE transcripts are required to induce silencing, it is possible that a certain level of active marks is present in the initial stages of silencing and that they are removed by erasers to allow for the deposition of H3K9 methylation and DNA methylation, as well as the silencing of TE loci, which are then stably maintained by repressive complexes. Alternatively, the process might be more dynamic, and the balance between active and repressive marks may need to be continuously maintained by the interplay between “erasers” and “writers”. Studying this interplay between histone “writers” and “erasers”, including histone methyltransferases and demethylases, in TE silencing could provide some answers to this question. Additionally, the role of TAD and 3D chromosome organization in TE silencing is just beginning to be explored, as is the issue of whether 3D conformation affects integration sites. Similarly, it will be important to determine the impact of TE integration on local folding. 

There are more than 1000 classes of TEs present in many copies, and some studies show that they are differentially regulated and can harbor distinct sets of chromatin marks. Nevertheless, which combination of marks is present in which context in a given specific TE is unclear. Importantly, deciphering the language behind the complex of epigenetic marking of TEs will provide insights into the regulatory mechanisms governing this still “dark” part of the genome. One major barrier to the bioinformatic analysis of TE sequences is their repetitive nature [211]. Advances in genome-wide technologies, as well as the development of new experimental and computational strategies to solve the longstanding issue of TE mapability, have the potential to further expand our understanding of the role of chromatin in TE regulation and shed light on the biological impact of TEs in eukaryotic genomes. Furthermore, the development of single-cell RNA-Seq (scRNA-Seq) strategies has allowed for the monitoring of gene expression at single-cell resolution, providing a powerful tool to observe cell activity and study cell-to-cell heterogeneity. However, in the majority of studies performed using scRNA-Seq technologies, TEs have been overlooked. Recently, two new algorithms were developed that can quantify TE expression in scRNA-Seq datasets [212,213]. Using these tools, the authors found that many TEs are specifically expressed in different cell types not only during embryonic development but also in mature somatic cells [212,213]. Developing tools to analyze the pattern of expression of TEs across different cell types and developmental stages will be essential to establish the contribution of TEs to cellular heterogeneity and to disease. 

In recent years, it has become evident that TEs become derepressed and active in many diseases, including cancer and neurodegenerative disorders. In cancer cells, increased TE expression and reactivation correlates with the loss of repressive chromatin modifications [214]. TE transposition can cause new mutations, as shown in colon cancers, where LINE1 insertions were found to compromise the function of the tumor suppressor adenomatous polyposis coli (APC) [215,216]. TE transcription can also induce the activation of neighboring oncogenes, a process known as onco-exaptation. A large-scale study examining RNA-Seq datasets from more than 7000 tumor samples revealed at least one onco-exaptation event in half of the tumor samples [176]. In addition to disrupting the sequence and/or expression of tumor suppressor genes and oncogenes, TE reactivation and derepression can affect the host genome in other ways. Expression of TE-derived proteins, such as ORFp1, a protein that controls LINE-1 retrotransposition, has been observed in many cancers, although the precise role of these proteins in tumorigenesis has not been established [59]. Additionally, LINE-1 and LTR can be the source of double-stranded RNAs (dsRNA) that can induce gene silencing [217]. Interestingly, it has been documented that TE-derived transcripts can activate the interferon response [23], and this property can be exploited to boost immunotherapy. Specifically, studies have shown that the use of epidrugs, such as DNMT inhibitors or LSD1 inhibitors, can induce TE expression, resulting in activation of an immune response, and that the combination of these epi-drugs with immunotherapy results in increased death of cancer cell [23,24]. Similarly, TE activation has been observed in neurodegenerative disorders. Interestingly, TE activation is often observed in healthy brains and has been linked to the diversification of the neuronal cell population [218]. However, TE activation seems to increase with age, especially in patients with neurodegenerative diseases [219,220]. According to the “transposon theory of aging“, a reduction in the cell defense mechanisms and a loss of heterochromatic marks during aging results in increased TE activation. An increased understanding of the epigenetic mechanisms underlying TE regulation can be expected to encourage the exploration of novel therapeutic avenues employing epidrugs for cancer or other human diseases.

## Data Availability

Not applicable.
